# Cross-cultural child and adolescent psychiatry research in developing countries

**DOI:** 10.1017/gmh.2015.8

**Published:** 2015-05-19

**Authors:** O. Atilola

**Affiliations:** Department of Behavioural Medicine, Lagos State University College of Medicine Ikeja, LagosNigeria

**Keywords:** Culture and mental health

## Abstract

Mental disorders are currently a major source of morbidity among children and youth globally. The bulk of the epidemiological data about childhood mental health morbidity currently comes from the industrialized countries which paradoxically host a small (about 20%) proportion of global children and youth population. As the world seek to generate more data on the mental health of the teeming children and youth population in low- and middle-income countries (LMICs), cross-cultural issues need be considered. This consideration is imperative for reasons which include the high level of ethno-diversity in LMICs; the contextual issues in the conceptualization of normal (and abnormal) childhood across cultures, the cross-cultural nuances in risk and protective factors, and the plurality of nature and expression of childhood psychopathology. As much as it is imperative to do so, advancing cross-cultural child and adolescent research in LMICs will need to overcome challenges such as inclusive sampling and cultural validation of instruments developed in the industrialized countries of the West. Funding, technical resources, and publication bias are other potential challenges. These issues are appraised in this narrative review and some ways forward are proffered.

## Introduction

The nature and appreciation of child health is changing, and one of the new paradigms is an increased appreciation of mental disorders as a source of childhood morbidity (Palfrey *et al*. 2005). This development is connected with the increasing appreciation of childhood mental disorders as major sources of distress to children, and a huge cost to society (Simpson *et al*. 2005; Smit *et al*. 2009). The appreciation of the increasing global burden of childhood mental disorders is however largely based on epidemiological surveys conducted largely in developing countries of Europe and North America (Merikangas *et al*. 2009). This situation is partly as a result of paucity of epidemiological and service research in the field of psychiatry (including child and adolescent psychiatry) in developing countries (Roberts *et al*. 1998; Patel & Sumathipala, 2001; Saxena *et al*. 2006).

Yet, the demographics of the global human population is skewed in such a way that most (up to 80%) of world children and youth live in low- and middle-income countries (LMICs, United Nations, 2003). This fact in itself argues in favor of developing a robust base for child and adolescent mental health (CAMH) epidemiological and service research in LMICs. Moreover, epidemiological research provides the scientific basis for estimates of the burden of diseases while service research dictates the strategies and plans to address the disease burden. As much as CAMH issues is a stated priority of World Health Organization in LMICs (World Health Organization, 2003); lack of indigenous CAMH epidemiological and service research in those regions will dictate that intervention policies are based on intuition or borrowed policies. To this extent, there is need for bridging the current gap in CAMH epidemiology and service research.

Child and adolescent psychiatry research has developed tremendously in the industrialized countries of the West (Costello *et al*. 2006; Merikangas *et al*. 2009), and have inadvertently set the global direction for empirical and theoretical frameworks for research in the field. In other words, the industrialized countries of the West have already set the direction for CAMH research, based on their own appreciation and understanding of the subject. The LMICs have and would still benefit from the framework already laid down as CAMH research in the region seek to expand. Research of any kind in the field of mental health must however be cognizant of cultural nuances (Groleau & Kirmayer, 2004; Halliburton, 2004) if such will not end up being de-contextualized or misleading. This is because culture not only shapes the programming of the mind (Heine, 2008), it influences several aspects of mental distress ranging from idioms of expression to the meaning of symptoms (Weisz *et al*. 1997, 2006; Canino & Alegria, 2008). This fact has to be factored into any effort at bridging the CAMH research gap in developing countries.

This conceptual review discusses the imperatives of and ways to develop cross-cultural CAMH research in LMICs. The challenges of CAMH research in LMICs and ways to address such are equally discussed.

## In context: what are the peculiarities of LMICs?

**Fig. 1.**
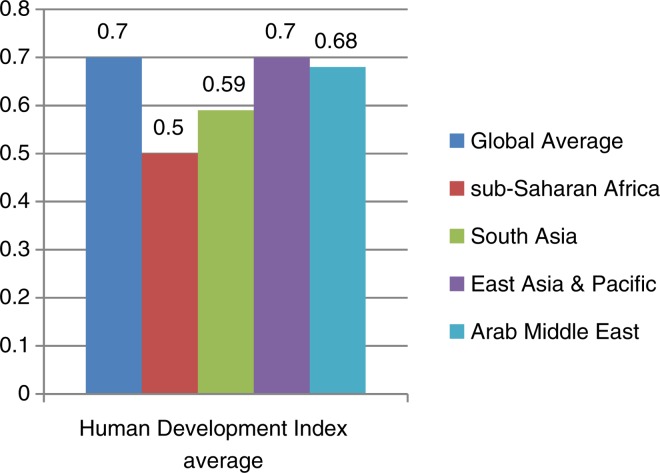
Comparison of the Human Development index from low- and middle-income regions with the global average. Data sourced from United Nation Development Programme (2014).

**Fig. 2.**
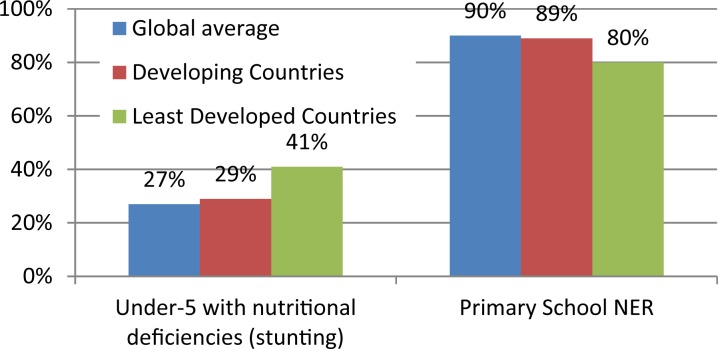
Social indicators for children (under-5 nutritional deficiencies and primary school NER) in developing and least-developed countries compared with global average. NER: Net Enrolment Ratio. Data sourced from UNICEF (2012).

**Fig. 3.**
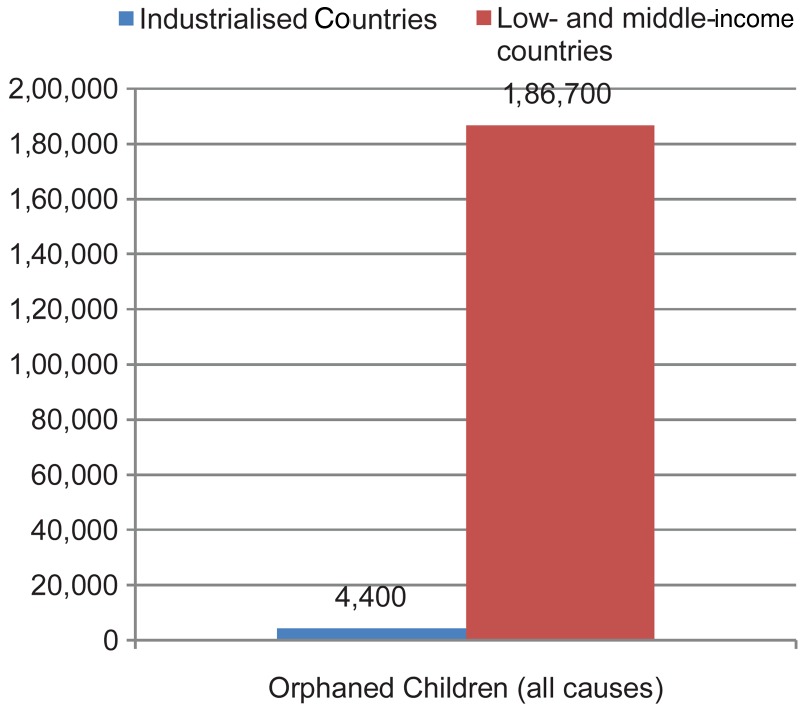
Social indicators for children (number of children orphaned) in low- and middle-income countries compared with global average. Data sourced from UNICEF (2012).

The LMICs host about 80% of the world children and youth population, apparently because of the high birth rate and lower life expectancies in these regions (United Nations, 2003). In this discourse, sub-Saharan Africa, South-East Asia, Southern America, Eastern Europe and parts of the Middle East is of particular reference as examples of regions populated by LMICs. These regions have the worst comparative social indicators for children and youth. For instance, compared with the industrialized countries or the global average, the LMICs fared worse in key indicators like Human Development Index, primary school Net Enrolment Ratio, under-five malnutrition, and others (see Figs. 1–3). Aside these poorer social indicators, these regions of the world are also prone to conflicts and political instability. In such setting, children and youth face a lot of biological, social, economic, and political challenges which may impact on their mental health and wellbeing.

Despite the apparent risk to CAMH in these regions, they are among the least represented in CAMH epidemiology and service research (Roberts *et al.*  1998; Patel & Sumathipala, 2001). Child and adolescent mental health policies in such regions are still largely driven by universalistic or globalized (Western) approaches. By reason of their cultural proximity, the industrialized countries of the world often share similar strategy and approaches in dealing with a wide range of social-economic and political issues. These ‘Western’ approaches often become hegemonic and perhaps inevitably transferred to the non-Western developing regions with weaker systems. Unfortunately, these two regions (industrialized Western and non-Western LMICs) are traditionally different in their socio-cultural milieu and social value-system. While the dominant industrialized countries of the West (example Australia, Canada, the USA and Great Britain) share the values of individualism, secularism, and internal locus of control; the LMICs (of Africa, South Asia, South America, Middle East) lean more towards external locus of control, strong family ties, deference to authority, and strong religious beliefs (Rosenthal, 1999, 2000; Inglehart & Welzel, 2011).

This basic polarity in cultural leaning and value systems have implications for globalized systems of assessment and intervention for a deeply cultural issue like CAMH. The globalized and universalistic view of CAMH systems have been challenged by empirical evidence which showed that childhood psychopathology operates differently across cultures (Wakefield *et al*. 2002; Roberts & Roberts, 2007). To compound this challenge is the fact that the LMICs are also the most culturally diverse. Global mappings of ethno-cultural diversity, for instance, have shown that the LMICs of the Amazon Basin, Central Africa, Indo-Malaysia, French Guiana, Suriname, and Guyana ranked highest in global ethno-cultural diversity (Loh & Harmon, 2005).

Therefore, CAMH researchers working in LMICs are confronted daily with dearth of indigenous epidemiology and service research to take action, and the stark burden and plurality of CAMH issues. They are also faced with the dilemma of not wanting to impose de-contextualized ‘Western’ standards of measure and intervention on one hand, and the expediency of doing something with what is at hand about an obviously unmet-need on ground. This situation provides a rationale for an urgent need to generate more culturally-nuanced epidemiological and service research in the field of CAMH in LMIC.

## Towards a culturally-nuanced child mental health research in LMICs

### Contextualized childhood

The prime contextual issue which cross-cultural and culturally-nuanced CAMH research in LMICs must be cognizant of is the fact that the concept of *childhood* is context-specific. The global knowledge and understanding of *childhood* has been largely shaped by the Western ideals. This ‘Westernized’ understanding of childhood has been introduced to the LMICs through the policies and activities of international agencies and NGOs working with children in those regions (Penn, 2002; Robinson, 2008; Ansell, 2010). This globalized concept of childhood is often taken as the ideal against which the normality or otherwise of childhood experiences in LMICs are adjudged.

Recent scholarly analyses have however revisited the universalistic assumptions about childhood by demonstrating that childhood is shaped by different social, economic, cultural, and historical contexts (Christensen & James, 2000; Holt & Holloway, 2006). The socio-cultural milieu within which the current Western concept of childhood emerged is different from those shaping the understanding of childhood in LMICs of the Global South (Hollos, 2002). One illustrative point will suffice to buttress the inevitability of plurality of childhood experiences: The family unit and by extension the nature of parenting is one of the most important of the several other operative environments for the creation of contextualized childhood (Hutchby & Moran-Ellis, 1998; Tamanovic, 2004). Yet, parenting itself is a function of the accumulated cultural experience of parents (Harkness & Super, 1996), and the cultural experience of parents is not universal in its nature and dynamics across cultures.

Just like it has influenced the understanding of childhood psychopathology (Timimi & Leo, 2009), the contextualized notion of childhood has found application in everyday issues of interest in CAMH. For instance, research has found cross-cultural nuances in, but not limited to, childhood sexuality (Nieto, 2004), child sexual-abuse (Pasura *et al*. 2013), child social-value systems (Genyue *et al*. 2011), and child play (Lee & Wohn, 2012). In other words, the concept of childhood lies at the intersection of multiple frames of reference (Pasura *et al*. 2013). The resultant definition of childhood in various contexts dictates to a large extent the way in which other ancillary concepts such as CAMH is also conceptualized and understood. Therefore, awareness and making sense of possible multiple models of childhood which inform the construction of CAMH and its related constructs, will definitely add much value to cross-cultural interpretation of CAMH research in developing countries.

### Diverse and contextualized CAMH risk and protective factors

The mental health of children and youth is shaped by a wide range of risk and protective factors which cuts across many layers of the childcare ecosystem (Berk, 2006). These layers will include families, peers, schools, neighborhoods, environments, local culture and practices, as well as local and international policies on childcare and child protection. The future of psychiatry (including child psychiatry) epidemiology lies in further understanding of how multiple and diverse risk and protective factors interact over time to produce psychopathology (Weich & Araya, 2004; Schwartz & Susser, 2006). Understanding the mixture and dynamics of the multi-level factors which influence CAMH is even more germane in LMICs where such factors are particularly diverse and plural. Therefore, this is one area which has to be borne in mind as CAMH epidemiological research in developing countries seeks to expand. Recent research has evolved theoretical models which can serve as operative frameworks for capturing the wide and intertwined risk and protective factors for CAMH in LMICs (Atilola, 2014).

In addition, the understanding of CAMH risk and protective factors need to be contextualized. It is not a deniable fact that social indicators for children are poorer in LMICs compared with the industrialized countries of the West. A cursory look at Figs. 1–3 as well as Unicef's State of the World's Children Data (Example: UNICEF, 2012) will establish this fact. As a result of this, the world had continued to view children in LMICs as living a deviant childhood which is riddled with physical- and mental-health risks. De-contextualized conception of childhoods had continued to fuel the conceptualization of childhood experiences in developing countries in terms of ‘risk’ and hardly from what they may uniquely have in a positive sense. Research in the field of resilience has shown that childhood ‘risk’ is a context-dependent factor (Rutter, 2006; Ungar *et al*. 2013). What may be classified as a risk factor from globalized conception of ‘risk’, which derived from research and understandings from industrialized countries of the West, could indeed be one of the sources of resilience among children in LMICs (Ungar *et al*. 2013).

A few illustrative examples will suffice to buttress these points. Firstly, in the context of LMICs where there is high level of unemployment (especially among women), mothers spend more time with their newborn children and infants. In the same setting, early childhood (pre-primary) education enrolment rate is lower compared with the industrialized countries of the West (see UNICEF, 2012). While lack of access to stimulating early-childhood education is a CAMH risk in industrialized countries, indigenous childcare resources will compensate for same in LMICs. Secondly and in the same vein, in contexts where formal schooling is relatively inaccessible, what is conceived as child labor may not only be socially acceptable, it may constitute an alternative way of children acquiring needed socialization; expressing their agency, deriving a feeling of self-efficacy, securing access to financial resources, and building resilience in the face of adversity (Liborio & Ungar, 2010). Thirdly, whereas the pursuit of a hobby can be an indicator of individual wellbeing and resilience in industrialized countries of the West (Brooks, 2006), same will not be a good measure of wellbeing in the lower-income countries. Rather, spending time working in the family's subsistence business may serve same purpose as pursuing a hobby for a child in developing countries.

Furthermore, it will be unreasonable to use family vacation as a measure of family wealth in lower-resourced regions where vacation is a luxury, but where families have a different view about leisurely use of family resources or indeed have devised other means of leisure and bonding outside of the globalized conception of leisure. This example is well illustrated in the Family Affluence Scale (Boyce *et al*. 2006), a globalized measure of family affluence. Atilola *et al*. (2013) has pointed out that most of the items in the Family Affluence Scale such as ‘car ownership’, ‘bedroom sharing’, and ‘holidaying’ may operate differently in measuring the concept of affluence in the multicultural LMICs . Fourthly and probably most illustrative of the contextualized nature of risk and resilience factors is the observation that children who migrate from low-resourced countries to industrialized countries of the West experienced a decline in their mental health as they drop their culture and values to adopt that of the host country (Berry *et al*. 2006).

In addition, research in the field of resilience has found that the experience of adversity does not always constitute a risk *sui generis*, but a function of the nature of the environment within which the adversity is operating (Rutter, 2006). In the face of chronic adversity such as poverty and chronic traumatic experiences, children over time may develop adaptive coping mechanism which translates to resilience in the long term (Masten, 2001; Masten & Narayan, 2012). This positive outcome is more likely when the micro-system (family, peer relations) of the child remains fairly stable despite adversity (Bonanno *et al*. 2011). This form of positive outcome has been termed *emergent resilience,* and it is akin to *minimal-impact resilience* which arises when acute traumatic events are not severe enough to engender a risk (Bonanno & Diminich 2013). In other words, when the micro-environment is right, chronic adversity can actually morph into a protective factor, same for low-threshold traumatic events.

Therefore, as much as children in LMICs may be experiencing a lot of adversity and supposed risks to their mental health, epidemiological, and service research in these regions will probably espouse more value if they adopt a contextualized view of these ‘risks’ rather than a wholesale adoption of the notion of ‘risk’ from the industrialized countries of the West. In other words, as much as socio-demographic and clinical correlates are important in service planning, a nuanced understanding of ‘risk’ and ‘protective’ factors for child mental health in LMICs will provide the right template for culturally relevant interventions. Consequently, more research that can deepen the understanding of those peculiar practices, activities, and relationships which constitutes coping mechanism, as well as which can explain the contextual meaning of otherwise adverse circumstances among children in LMICs are needed.

### Cross-cultural variability of childhood psychopathology

Cultural factors affect different aspects of the mental health of children, including idioms of expression and preferred mode of treatment (Weisz *et al*. 2006; Canino & Alegria, 2008). There is research evidence that certain forms or syndrome of psychopathology are also existent in some cultures but not in another (Weisz *et al*. 2006). Other earlier observation include that certain childhood psychopathological phenomena are culture-bound as they were observed almost exclusively in a particular region. While the global nosology of psychiatric disorders has endeavored to capture some of these unique mental phenomena, others are yet to form part of the global psychiatric categories.

Interestingly, many of the well-known culture-bound psychopathological phenomena have been described among children and young persons in LMICs. For example, *ataques de nervios* – a culture-bound panic-like disorder – has been described among adolescents and adults of hispanic descent (Keough *et al*. 2009). Another syndrome called *nervios* which is believed to be a culture-bound syndrome of reactive depression has been described among children who suffered abuse or neglect in the Ecuadorian Andes (Pribilsky, 2001). *Brain-fag* syndrome, a culture-bound syndrome of heat sensation around the head; inability to concentrate while reading, and general fatigue, has been reported almost exclusively among children and youth in Nigeria and other parts of Africa (Ola *et al*. 2009). Furthermore, *cen* – a trauma related psychological disorder which is similar to but distinct from posttraumatic stress disorder – has been described among child-soldiers in Uganda (Neuner *et al*. 2012). *Ascetic syndrome,* which is characterized by social withdrawal, severe sexual abstinence, practice of religious austerities, lack of concern with physical appearance, and considerable loss of weight has also been described exclusively among adolescents in India (Bhatia, 1999). *Ogbanje* and *emere* are other psychopathological syndromes which have been described among Nigerian children but which are yet to be captured in global psychiatric nosology (Ilechukwu, 2007).

Therefore, efforts to expand epidemiological studies of CAMH in LMICs should be aware of cultural peculiarities in psychopathology. It is also important to acknowledge that aside the global nosology of child and adolescent psychiatric disorders; there are many other CAMH phenomena within the diverse ethnographic landscape of LMICs.

## Setting the agenda: advancing cross-cultural child-psychiatry research in LMICs

### Epidemiological research

In as much as there is a genuine need to expand CAMH in LMICs so as to bridge the current gap, cross-cultural considerations should be an integral part of such effort. Cultural contextualization should be incorporated into the entire research process from the conceptualization stage to the final stage of interpreting the results (Hughes *et al*. 1993; Monteiro & Balogun, 2014). At the heart of conceptualization of research is the determination of the methodology, including the sample population and the measures or instruments to be used. However, identifying the appropriate sampling technique for cross-cultural validity and comparability can pose a challenge. This challenge is at the heart of the complex dichotomy of *emic* and *etic* approaches in cross-cultural psychological research. For a long time, the dynamics of cross-cultural psychology had derived from the basic dilemma between the *etic* and the *emic* approaches (Berry, 1989; Berry *et al*. 1992; Helfrich, 1999). The *etic* approach emphasizes the universality of psychological phenomena and seeks to establish or refute this in other settings while the *emic* dwell on the uniqueness of such phenomena in different cultures.

During sampling in CAMH research, one of the challenges of adopting the *etic* approach is that CAMH as an attribute-of-interest co-varies with other variables (such as parenting style, local understanding of childhood, and other ecological environment of the child) which also vary from one region to another. Attempt to control or eliminate interfering variables with special sampling techniques will eventually lead to atypical sampling in one of the regions and subsequent confusion (Helfrich, 1999). In other words, closely matching children-sample from LMICs with those from high-income countries in an effort to achieve cross-cultural comparability will inadvertently result in comparing typical subjects from one culture with an atypical group from another (Bose & Jennings, 2005). The *emic* approach tries to compensate for the culture-boundness of other interfering variables by giving reference to individual and regional uniqueness. The danger with the *emic* approach lies in the arbitrariness of emphasizing cultural relativism which tends to do away with the very idea of cross-cultural comparability (Helfrich, 1999). Unifying theoretical frameworks has been proposed to overcome the weaknesses of both *etic* and *emic* approaches and advance cross-cultural psychology research (Helfrich, 1999), the discussion of which is outside of the scope of the present paper.

Illustrative of the foregoing is the situation whereby, unlike in the industrialized countries of the world where school enrolment can be as high as 100%, the rates in some LMICs countries can be as low as 60% (UNICEF, 2012). Therefore, while school-based samples can be conveniently used to conduct representative CAMH research in the high-income countries, same method will exclude a large proportion of children in LMICs and thereby limiting cross-cultural comparability from the outset. Aside this, the socio-demographic characteristics of school-children and the educational systems varies from one country to another. The use of house-hold surveys may address the challenge of school-enrolment in LMICs but it will throw-up the challenge of excluding high-risk and difficult-to-reach groups like street-children, institutionalized children, and other children in out-of-home settings who are often in large numbers in LMICs. Use of household-sampling to achieve cross-cultural comparability between high-income countries and LMICs will also have overlooked other ‘*organismic variables’* (Edwards, 1971) such as differences in teaching styles, child-rearing methods, and ecological differences.

These are complex issues which can hardly be resolved in any simplistic way. As such, sampling techniques for cross-cultural comparability and validity is still a hotly debated issue in cross-cultural social-science research (Zheng, 2013). There is hardly any ideal approach at present which allows a uniform sampling method in all regions. This is almost unachievable because a sampling frame which includes all individuals of the target population does not necessarily exist in each country (Zheng, 2013). There is however some research evidence that probability sampling techniques in general generates results that allow cross-cultural comparability and validity better than non-probability sampling (Zheng, 2013). However, it is still advisable that every cross-cultural CAMH research examines the uniqueness of the index study and the resources available in determining which method to adopt.

*Etic* and *emic* issues also find application in other areas of the research process beyond sampling. In the area of measures to use for the survey, a large number of standardized diagnostic/ screening instruments for general psychiatric disorder (Sartorius & Janca, 1996) and specific child psychiatric disorders (Verhulst & van der Ende, 2006) has been developed. Many of the child psychiatry measures are however developed from Western cultures and are yet to be validated and adapted for use in LMICs (Canino & Alegria, 2008). The paucity of culturally validated child psychiatric measures has been observed and lamented in systematic reviews of CAMH epidemiological surveys from LMICs (Cortina *et al*. 2012). For cross-cultural validity, *etic* principles dictates that measures that are developed from elsewhere are validated by adapting such to the local culture, taking cognizance of cross-cultural issues which may arise especially in the course of translation and contextualization of such measures.

Translation of standardized child psychiatry measures into the local language is a task which a CAMH researcher in the multilingual LIMCs of the Global South will often have to carry out. Rigorous translation and back-translation alone may not guarantee equivalence in meaning, as the same words may have different associations among children and adolescents in different cultures (Bose & Jennings, 2005). There had been reports of spurious results for child psychiatric conditions which were as a result of conceptual misinterpretation of a large number of quantitative questions by children and adults in LMICS (King & Bhugra, 1989; Gjersing *et al*. 2010). Researchers should be aware of this potential pitfall in the translation process. After translation and back-translation, cognitive debriefing of a sample of children and adolescents is needed to explore comprehensibility, response process, clarity, appropriateness, and to account for or correct any problem with conceptual meaning or ambiguity in each of the item of the scales. This method has been introduced into validation of child psychiatry measures in LMICs (Atilola & Stevanovic, 2014). This will indirectly improve the construct validity of the translated measure.

Aside the issue of translation, *emic* principles recognizes the cross-cultural variations in the conceptualization and the idiom of expression of mental disorders (Weisz *et al*. 2006; Canino & Alegria, 2008). This fact speaks to a need for rigorous contextualization of instrument and measures in other areas such as construct, conceptual, and criterion validity. Failure to ensure equivalence in all these areas and more may lead to misclassification, distortion of prevalence estimates, and severe limitation of cross-cultural comparability of findings. The processes involved in achieving validity of an instrument in various dimension is beyond the scope of the present discourse but is available elsewhere (Matias-Carrelo *et al*. 2003). Furthermore, in regions where culture-bound syndromes have been established, items which can assess for these special conditions can be added to standardized measures (Canino & Alegria, 2008) and thus improving content validity.

Despite careful and rigorous translation and validation, the limitation of quantitative measures in capturing the complexity of human behavior, emotion, and experience is well documented in the literature (Guba & Lincoln, 1994; Groleau *et al.* 2006). Therefore, in multicultural settings, qualitative research is best suited for understanding the cultural nuances of mental health beyond the ‘categorical’ information obtained through quantitative interview. It also presents opportunities to obtain rich information from populations in LMICs whose literal and broader symbolic voices are often underrepresented in global mental health understanding and research (Monterio & Balogun, 2014). Therefore, when the constraints of cost and logistic permits, as much as possible, *emic* ideals dictates that qualitative aspect should be included in CAMH research in LMICs either as the sole method or mixed with quantitative items.

Another key step in the research process that needs to be culturally nuanced is the interpretation of results. The findings of a research and the information deduced from same needs to be properly contextualized. Without a conscious effort at restraint, there is always a tendency for CAMH researchers in LMICs (who are often exposed to globalized ideas about CAMH in their training) to be swayed by their training while interpreting results. Let us take for instance, the interpretation of risk and protective factors. De-contextualized understanding of results may lead to irrelevant conclusion or recommendations. One example suffices to illustrate this concern: On the strength of a finding that larger family size and lower parental socio-economic status was ‘associated’ with depressive symptoms among school children in Nigeria, Fatiregun & Kumapayi (2014) concludes that these factors constitute ‘risks’ and recommended that ‘*family planning with focus on reducing family size and poverty alleviation interventions, especially among household…. could be long-term measures in reducing depressive symptoms among adolescent*’! This conclusion did not take into account the theoretical concept of intersectionality which dictates that within a cultural setting, the multiple factors which contribute to human social-outcomes intersect and interacts in a simultaneous, inseparable, and intertwined manner (McCall, 2005). The conclusion also is unmindful of the fact that large family size may have its own inherent advantages for personal and family dynamics. Such view may have been influenced by the idealized model of nuclear family from the West. A conclusion which takes into account the cultural meaning of ‘large family size’ and the contextual definition of ‘poverty’ would have being more nuanced.

Finally, it is important to note that all the efforts at conducting culturally-competent CAMH research in developing countries will come to naught if the finished product, in this case the manuscript, are not published. Earlier research has found that there is a gap in the presence of developing countries in global psychiatry literature (Saxena *et al.* 2006; Patel, 2007). This situation is as a result of relatively lower number of submissions from developing countries, and even much lower number of submissions which scaled beyond editorial screening to go for peer review (Kornadsen & Munk-Jorgensen, 2007). The reasons for this situation are multifaceted and include limited access to information and advice on research design and analyses; language barrier, material and financial limitations, and policy constraints (Bulletin of the World Health Organization, 2004). Other more intricate barrier is the imbalance and inequity in the composition of editorial boards of international health journals (Mohammadi *et al*. 2011). This has led to a limited appreciation by editorial boards of the research needs of, and realities in, LMICs with attendant negative impact on publication of mental health (including CAMH) research in the region (Bulletin of the World Health Organization, 2004). The same challenge of decision bias has also been observed with reviews of grant applications for mental health research coming from LMICs (Jorm *et al*. 2002).

Most of these problems can be addressed through capacity building and training. The need to improve research infrastructure and capacity in LMICs has been severally highlighted (Saxena *et al*. 2006; Patel, 2007). Research training partnerships with support from high-income countries are critical to building research capacity in LMICs, and there are several successful models of such (Patel, 2007; Thornicroft *et al*. 2012; Sweetland *et al*. 2014). It is often argued that funding of mental health research in general is not a priority of international donor agencies (Alem & Kebede, 2003). However, mental health research often features on the priority list of big health funders such as National Institute of Health (NIH), Global Fund, Bill & Melinda Gates Foundation (Ravishankar *et al*. 2009). The problem appears to lie in relative underfunding based on disease burden. Gillum *et al*. (2011) analyzed the research funding of the NIH and found that infectious diseases and physical non-communicable diseases (Example: Diabetes) do receive far more funding compared with mental health conditions based on their relative disease burden. The funding gap is even wider for CAMH research as only a small fraction of what was available for mental health research in LMICs trickles into CAMH research (Bakare *et al.* 2014). The imperatives of extending parts of global mental health research funding to the area of CAMH has been emphasized (Bakare *et al.* 2014).

One of the most important areas of focus of such funding should be capacity building. This is because without capacity to utilize effectively, these funds will yield little value (Patel, 2007). Capacity building which is situated within LMICs but with foreign funds are generally better. This is because the training/capacity building stands a chance of being contextualized and reduces the risk of brain-drain. Home grown capacity building programs like the Master degree program of the Center for Child and Adolescent Mental Health (CCAMH) in Nigeria where research capacity is being built locally with the support of MacArthur Foundation is an ingenious example (Omigbodun, 2014). In the same vein, global research funds should support the growth of local psychiatry and CAMH journals which will give priority to local content in both their editorial and review process.

After all said and done, funding of cross-cultural CAMH research in developing countries cannot always be guaranteed. There is need to evolve more cost effective and efficient ways of conducting quality research. One way of achieving this is through collaborative research. In low-resource settings, collaboration allows for resource (material and technical) sharing, shared responsibilities, and cost-sharing. The International Child Mental Health Study Group- a consortium of young and talented CAMH researchers from developing countries – have trailed the blaze on how this form of collaboration can advance cost-effective cross-cultural CAMH research (Franic *et al*. 2014). More importantly, there is need to mobilize low LMICs to take more ownership in defining their own research policies, rather than merely being passive recipients of international aid for research and development (Rudan, 2012).

### Service research

Child and adolescent mental health service research should necessarily complement epidemiological research in LMICs. This is because one of the ends of epidemiological research is translation to service. Aside a need for cultural contextualization, CAMH service research in LMICs must set its own agenda based on the peculiarities and, of course, limitations within the region. A key step in this direction is to avoid wholesale importation of programs and strategies from high-income countries without cross-cultural considerations. For example, many of the resilience-enhancing life skills such as coping-skills and assertiveness in the Western literature are known to be context specific (Ungar, 2008). Therefore, what promotes child and youth resilience in LMICs may be different from those from the globalized understanding of life-skills. A recent study in Uganda found that the coping strategies of youth in the country during adversity are distinct from that of Western countries, and that youth mental health intervention programs which are based on Western understanding may in fact be counterproductive (Betancourt *et al.* 2011). Research into culturally-appropriate resilience-enhancing skills of children and youth in LMICs are needed, as they will form the basis of culturally-relevant and effective CAMH promotion programs.

Similarly, the efficacy of psychological treatments for common mental disorders among children and adolescents has been established in systematic reviews (James *et al*. 2013) and so is their safety ahead of pharmacological treatments (Picouto & Braquehais, 2013). Psychological interventions such as cognitive-behavioral therapy derive from understanding of the social cognition which drives thinking and behavior. However, the cognitive processes that underpin thinking, behavior, and motivations are not universal as socio-cultural factors have been recognized as an important modulator of cognitive processes (Nisbett & Norenzayan, 2002). Therefore, the efficacy of psychological therapies that are developed in the West in the LMICs will always remain suspect. There has been some attempt at adapting some of these psychological interventions for childhood psychopathology to the local culture in some LMICs (Murray *et al*. 2013). The main thrust of such adaptations had been to identify areas of universal applicability and necessary modifications to enhance cross-cultural acceptability. More of such researches are desperately needed.

In the same vein, it is known that the unique understanding of CAMH and disorders in LMICs especially in Africa has created a situation whereby a significant number of children in need of CAMH services seek alternative mental health services (Bell *et al.* 2001; AbdulMalik & Sale, 2012; Bakare, 2013). The role of alternative mental health services in reducing mental health morbidity has been established in the literature (Stradford *et al*. 2012). The place of alternative mental health services such as spiritual healing, faith healing, herbal treatment, and acupuncture (which are quite popular in LMICs) is yet to be fully evaluated among children and youth. Expanding service research to this area provides an opportunity to complement orthodox CAMH service which is ordinarily very constrained in LMICs (Robertson *et al.* 2010). Important directions in achieving this include local mapping of complementary/ and alternative services for CAMH disorders in different localities; establishing the efficacy and safety of these services, exploring the unique ethical and legal concerns for CAMH practitioners, and developing the framework for integrative care.

## Conclusion

There is a genuine reason to bridge some of the current gap in global CAMH research. The focus of this should be to increase the presence of data from LMICs. The fact that most of the LMICs are multicultural in nature calls for consideration of cultural nuances in the course of any effort geared towards achieving this. The pathway towards a culturally-nuanced CAMH research in LMICs will require a contextualized understanding of the concept of *childhood* in such regions relative to the high-income countries of the West. It will also require an understanding that CAMH risk and protective factors can be quite diverse and contextualized, and that childhood psychopathology may vary across cultures. There is also a need for culturally-nuanced intervention strategies. It is however pertinent for CAMH researchers in LMICs to keep pace with relentless globalization, and to follow and document the trends as cultural milieu in itself continue to be dynamic (Draguns & Tanaka-Matsumi, 2003).
